# Supplementary material for: Phosphorylation regulates tau’s phase separation behavior and interactions with chromatin
Source: Commun Biol. 2024 Mar 1;7:251. doi: 10.1038/s42003-024-05920-4 (PMC10907630; doi:10.1038/s42003-024-05920-4)
Supplement: Supplementary file 2 — Description of Additional Supplementary Files [file 42003_2024_5920_MOESM2_ESM.pdf]

Description of Additional Supplementary Files

File name: Supplementary Data 1

Description: The source data behind the graphs in the paper .
