# Supplementary material for: Phosphorylation regulates tau’s phase separation behavior and interactions with chromatin
Source: Commun Biol. 2024 Mar 1;7:251. doi: 10.1038/s42003-024-05920-4 (PMC10907630; doi:10.1038/s42003-024-05920-4)
Supplement: Supplementary file 4 — Reporting Summary [file 42003_2024_5920_MOESM4_ESM.pdf]

nature portfolio

Corresponding author(s): Debelouchina GT

Last updated by author(s): Feb 7, 2024

Reporting Summary

Nature Portfolio wishes to improve the reproducibility of the work that we publish. This form provides structure for consistency and transparency in reporting. For further information on Nature Portfolio policies, see our Editorial Policies and the Editorial Policy Checklist.

Statistics

For all statistical analyses, confirm that the following items are present in the figure legend, table legend, main text, or Methods section.

n/a	Confirmed	
☐	☒	The exact sample size (*n*) for each experimental group/condition, given as a discrete number and unit of measurement
☐	☒	A statement on whether measurements were taken from distinct samples or whether the same sample was measured repeatedly
☐	☒	The statistical test(s) used AND whether they are one- or two-sided<br>*Only common tests should be described solely by name; describe more complex techniques in the Methods section.*
☒	☐	A description of all covariates tested
☐	☒	A description of any assumptions or corrections, such as tests of normality and adjustment for multiple comparisons
☐	☒	A full description of the statistical parameters including central tendency (e.g. means) or other basic estimates (e.g. regression coefficient) AND variation (e.g. standard deviation) or associated estimates of uncertainty (e.g. confidence intervals)
☒	☐	For null hypothesis testing, the test statistic (e.g. *F*, *t*, *r*) with confidence intervals, effect sizes, degrees of freedom and *P* value noted<br>*Give P values as exact values whenever suitable.*
☒	☐	For Bayesian analysis, information on the choice of priors and Markov chain Monte Carlo settings
☒	☐	For hierarchical and complex designs, identification of the appropriate level for tests and full reporting of outcomes
☒	☐	Estimates of effect sizes (e.g. Cohen's *d*, Pearson's *r*), indicating how they were calculated

Our web collection on statistics for biologists contains articles on many of the points above.

Software and code

Policy information about availability of computer code

Data collection	Topspin 3.2 was used for NMR data acquisition.
Data analysis	ImageJ 1.53t was used to quantify band intensities from gel images. GraphPad Prism 9.5 was used for curve fitting of FRAP, EMSA, and fluorescence anisotropy. Microsoft Excel was used for basic analyses (averaging, etc). NMR spectra were processed with TopSpin 4.0.5 and analyzed using NMRFAM-SPARKY and CCPNMR 3.0.2. Nucleosome images with mapped perturbations were rendered using ChimeraX 1.4. MaxQuant 2.0.3.0 was used for mass spectrometry data analysis.

For manuscripts utilizing custom algorithms or software that are central to the research but not yet described in published literature, software must be made available to editors and reviewers. We strongly encourage code deposition in a community repository (e.g. GitHub). See the Nature Portfolio guidelines for submitting code & software for further information.

Data

Policy information about availability of data

All manuscripts must include a data availability statement. This statement should provide the following information, where applicable:

- Accession codes, unique identifiers, or web links for publicly available datasets
- A description of any restrictions on data availability
- For clinical datasets or third party data, please ensure that the statement adheres to our policy

All data are included in the manuscript, the supplementary information or as supplementary data. Our NMR data analysis is based on published chemical shifts available as BMRB Entry 27457. Processes NMR spectra are available on Zenodo: https://doi.org/10.5281/zenodo.10578886

Human research participants

Policy information about studies involving human research participants and Sex and Gender in Research.

Reporting on sex and gender	N/A
Population characteristics	N/A
Recruitment	N/A
Ethics oversight	N/A

Note that full information on the approval of the study protocol must also be provided in the manuscript.

Field-specific reporting

Please select the one below that is the best fit for your research. If you are not sure, read the appropriate sections before making your selection.

☒ Life sciences ☐ Behavioural & social sciences ☐ Ecological, evolutionary & environmental sciences

For a reference copy of the document with all sections, see nature.com/documents/nr-reporting-summary-flat.pdf

Life sciences study design

All studies must disclose on these points even when the disclosure is negative.

Sample size	N/A
Data exclusions	Several concentrations were excluded from EMSA fitting due to the difficulty of defining the exact gel band boundaries on the image. See caption for Figure 2b, which states: 177 bp DNA, 147 bp MN, and 177 bp MN were quantified in triplicate for the following data points: 0, 125, 250, 500, and 1000 nM. Earlier data were excluded due to the smearing and complex bands overlapping with the unbound probe.
Replication	We performed at least three biological replicates per experiment. The number of replicates for each experiment has been indicated in the relevant figure captions and/or methods section.
Randomization	N/A
Blinding	N/A

Reporting for specific materials, systems and methods

We require information from authors about some types of materials, experimental systems and methods used in many studies. Here, indicate whether each material, system or method listed is relevant to your study. If you are not sure if a list item applies to your research, read the appropriate section before selecting a response.

Materials & experimental systems

n/a	Involved in the study
☒	☐ Antibodies
☒	☐ Eukaryotic cell lines
☒	☐ Palaeontology and archaeology
☒	☐ Animals and other organisms
☒	☐ Clinical data
☒	☐ Dual use research of concern

Methods

n/a	Involved in the study
☒	☐ ChIP-seq
☒	☐ Flow cytometry
☒	☐ MRI-based neuroimaging
